# Light Intensity and Nitrogen Concentration Impact on the Biomass and Phycoerythrin Production by *Porphyridium purpureum*

**DOI:** 10.3390/md17080460

**Published:** 2019-08-07

**Authors:** Juan Eduardo Sosa-Hernández, Laura Isabel Rodas-Zuluaga, Carlos Castillo-Zacarías, Magdalena Rostro-Alanís, Reynaldo de la Cruz, Danay Carrillo-Nieves, Carmen Salinas-Salazar, Claudio Fuentes Grunewald, Carole A. Llewellyn, Eugenia J. Olguín, Robert W. Lovitt, Hafiz M. N. Iqbal, Roberto Parra-Saldívar

**Affiliations:** 1 Tecnologico de Monterrey, Escuela de Ingenieria y Ciencias, Campus Monterrey, Ave. Eugenio Garza Sada 2501, Monterrey, Nuevo Leon 64849, Mexico; 2 Tecnologico de Monterrey, Escuela de Ingenieria y Ciencias, Av. General Ramón Corona 2514, Nuevo México, Zapopan C.P. 45138, Jalisco, Mexico; 3 Department of Biosciences, Singleton Park, Swansea University, Swansea, Wales SA2 8PP, UK; 4 Environmental Biotechnology Group, Institute of Ecology(INECOL), Carretera Antigua a Coatepec No. 351, Xalapa, Veracruz 91070, Mexico; 5 College of Engineering, Swansea University, Swansea SA2 8PP, UK; 6 Membranology Ltd., Unit D5 Rainbow Business Centre, Swansea SA7 9FP, UK

**Keywords:** chemical stress, physical stress, pigments, microalgae growth, phycoerythrin

## Abstract

Several factors have the potential to influence microalgae growth. In the present study, nitrogen concentration and light intensity were evaluated in order to obtain high biomass production and high phycoerythrin accumulation from *Porphyridium purpureum*. The range of nitrogen concentrations evaluated in the culture medium was 0.075–0.450 g L^−1^ and light intensities ranged between 30 and 100 μmol m^−2^ s^−1^. Surprisingly, low nitrogen concentration and high light intensity resulted in high biomass yield and phycoerythrin accumulation. Thus, the best biomass productivity (0.386 g L^−1^ d^−1^) and biomass yield (5.403 g L^−1^) were achieved with NaNO_3_ at 0.075 g L^−1^ and 100 μmol m^−2^ s^−1^. In addition, phycoerythrin production was improved to obtain a concentration of 14.66 mg L^−1^ (2.71 mg g^−1^ of phycoerythrin over dry weight). The results of the present study indicate that it is possible to significantly improve biomass and pigment production in *Porphyridium purpureum* by limiting nitrogen concentration and light intensity.

## 1. Introduction

Phycoerythrin (PE) is a bioactive pigment mainly found in Rhodophyta, but it can also be recovered from Cyanobacteria [1]. This water-soluble pigment is a bioactive molecule with interesting properties such as anti-inflammatory, immunosuppressive, antitumor, antioxidant, antidiabetic, and antihypertensive effects [2,3,4,5]. In addition, PE moiety can be exploited as a fluorescent probe useful in cell tracking and imaging assays such as flow cytometry, fluorescent immunoassays, and immunophenotyping. [6,7]. As well as its application in the biotechnological and medicine areas, PE can be used in the food and cosmetic industries [8,9]. Commercial PE from microalgae has been produced by *Spirulina*, *Rhodella*, and *Porphyridium* [10,11,12]. The market price of this purified pigment is around $200 USD mg^−1^ (Sigma-Aldrich, product number: 52412-1MG-F), this being why PE is regarded as a molecule of high value [13]. *Porphyridium purpureum* has the ability to grow in different culture systems and locations [14], coupled with the capacity to produce and accumulate a large amount of PE, which production can be enhanced when the microalgae are cultured under stress conditions [11]. The effect of different physical and nutritional factors on the culture conditions of different microalgae groups has been investigated for several purposes, including the production of biomass, pigments, oil, and other metabolites [11,15,16,17,18]. Light intensity is one of the most critical factors that influence microalgal pigment composition. This is extremely important in those microalgae who have a complex harvest light system such as *Porphiridium* because, for instance, PE is the major accessory light-harvesting compound in Rhodophyta [19,20]. On the other hand, nitrogen is a fundamental constituent for protein synthesis, cell division and it also is required for the synthesis of photosynthetic pigments [21,22]. The effect of these two factors on *Porphyridium* strains has been reported in some studies [23,24,25]. However, in most of the cases, the variables of light intensity and nitrogen concentrations have been evaluated separately. Furthermore, most of the investigations regarding light influence on the production of PE focused only in the evaluation of different wavelengths [26,27] or illumination periods [28], but scarce information is related to light intensity.

Complementing biotechnology work, mathematical models have been widely used to explore microalgae growth. The application of computational power allows us to understand, predict and optimize cellular processes present in microalgae, without the need for experimental tests [29]. Mathematical models have been used in the cultivation of *P. purpureum*, however, because of the complexity of these models, it is difficult to apply them [30,31]. The Gompertz model allows us to predict parameters such as maximum specific growth rate (*µ_max_*), latency phase (*λ*), duplication time of microalgae cells (G), productivity and because of its simplicity, it can be easily applied; however, this model has not been applied in the growth of *P. purpureum* [32,33,34].

Due to the high value and remarkable diverse bioactivities of PE, in-depth exploration about the abiotic parameters management of microalgae cultures is needed in order to improve its production. In the present study, the effect of different light intensities and nitrogen concentrations were evaluated with the aim to obtain high biomass production and to accumulate a high PE concentration in *P. purpureum*. Besides the identification of the best levels of the abiotic factors evaluated, the correlation between the response variables was aimed as well.

## 2. Results

### 2.1. Experimental Results and Mathematical Model Fitness

The Gompertz model was used with the same conditions from the experimental results. In this manner, it was possible to get parameters that are not directly quantifiable. To fit this model, all the data were used in hours instead of days to have a better resolution in the model, then the time parameter was returned to days. The model was used to compare all experiments performed. The mathematical modeling to determine the biological parameters of *P. purpureum* culture, as well as the productivity and determination coefficient of the linear regression model obtained between the experimental and simulated results, are shown in Table 1.

The model fitness parameter used was the determination coefficient *R*^2^, which are over 90% for almost all curves. The two experiments under 90% were in the light intensity of 65 µmol m^−2^ s^−1^. Both experiments had 0.075 and 0.450 g L^−1^ concentration of NaNO_3_ and were in the range of 85% which is reasonably expected to be the biological variability of growth [35].

According to the productivity of the systems that were subjected to one way ANOVA analysis and paired two-sample mean with *t*-Test analysis, the values of productivity were higher (*p* = 0.105) for 65 µmol m^−2^ s^−1^ and significantly (*p* = 0.047) higher 100 µmol m^−2^ s^−1^ compared to the value registered at 30 µmol m^−2^ s^−1^, which were 350 and 386 mg L^−1^ d^−1^, respectively.

### 2.2. Influence of Light Intensity and NaNO_3_ Concentration on the Production of P. purpureum Biomass

**Figure 1:**
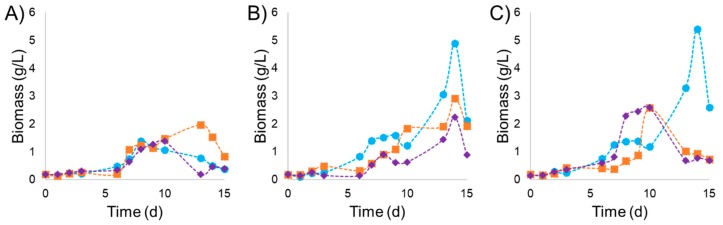
Biomass concentration of *P. purpureum* (g L^−1^) during cultivation under three different light intensities (**A**) 30, (**B**) 65 and (**C**) 100 (µmol m^−2^ s^−1^); and three different NaNO_3_ concentrations 0.075 (●), 0.225 (■) and 0.450 (♦) (g L^−1^). The error is less than 5% or in general, so it is in the range of the symbol size.

The kinetics of the biomass production from the culture of *P. purpureum* under different light intensities and NaNO_3_ concentration is presented in Figure 1A–C. The lowest biomass production was observed at 30 µmol m^−2^ s^−1^ regardless of the variation of nitrogen source (Figure 1A and Table 1). Under these culture conditions (30 µmol m^−2^ s^−1^), in the tests at 0.075 and 0.225 g L^−1^ of NaNO_3_, a delay in the latency phase of five days was observed, while at 0.45 g L^−1^ of NaNO_3,_ it was reduced to three days (also, according to the fit parameters shown in Table 1). This could be associated with the fact that the medium had a higher concentration of this nutrient. In addition, the maximum specific growth rate and productivity values were higher for the kinetics with nitrogen source of 0.075 and 0.225 g L^−1^ of NaNO_3_ and the duplication time of microalgae cells was shorter, compared with the condition of 0.45 g L^−1^ of NaNO_3_ (see Table 1).

Under a higher light intensity of 65 µmol m^−2^ s^−1^, a lag phase of six days and exponential phase of eight days of exponential were observed for the three concentrations of NaNO_3_ evaluated. Overall, the culture reached a maximum biomass concentration at day 14, with 4.896, 2.914, and 2.248 g L^−1^ for 0.075, 0225, and 0.450 g L^−1^ of NaNO_3_, respectively (see Figure 1).

Furthermore, high values of biomass were obtained under an increased light intensity at 100 µmol m^−2^ s^−1^ (Figure 1C). The maximum biomass production of 5.403 g L^−1^ was obtained at 14 days when the concentration of NaNO_3_ was fixed at 0.075 g L^−1^. The maximal production for the treatments with NaNO_3_ concentrations of 0.225 and 0.450 g L^−1^ were 2.579 and 2.598 g L^−1^, respectively at 10 days of culture. Culture conditions fixed at 100 µmol m^−2^ s^−1^ of light intensity and 0.075 g L^−1^ of NaNO_3_ concentration resulted in the highest productivity and maximum specific growth rate (μ_max_) with values of 0.386 g L^−1^ d^−1^ and 0.015 d^−1^**,** respectively. Similar productivity of 0.349 g L^−1^ d^−1^ was obtained under the concentration of NaNO_3_ 0.075 g L^−1^ by applying a light intensity of 65 µmol m^−2^ s^−1^. The productivity under a light intensity of 30 µmol m^−2^ s^−1^ was lower (and independent) than that of the NaNO_3_ concentration. The low levels of NaNO_3_ resulted in a biomass of 0.950 g L^−1^, while the biomass obtained under the high level of NaNO_3_ was 0.340 g L^−1^. In other words, 2.79 times more biomass was obtained maintaining of NaNO_3_ at a low concentration.

### 2.3. Influence of Light Intensity and NaNO_3_ Concentration on the Production of PE by P. purpureum

**Figure 2:**
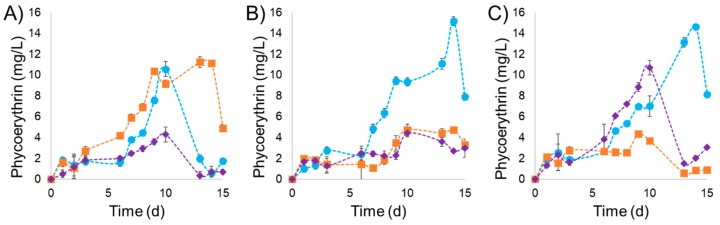
Phycoerythrin concentration (mg L^−1^) during cultivation under three different light intensities (**A**) 30, (**B**) 65 and (**C**) 100 (µmol m^−2^ s^−1^); and three different NaNO_3_ concentrations 0.075 (●), 0.225 (■) and 0.450 (♦) (g L^−1^).

The kinetics of PE production by *P. purpureum* under different light intensities and NaNO_3_ concentrations is presented in Figure 2. The experiments under a light intensity of 30 µmol m^−2^ s^−1^ are shown in Figure 2A. Under these culture conditions, the three experiments showed similar lag phases (6 days). A maximum PE concentration of 11.24 mg L^−1^ (5.70 mg g^−1^ PE/DW) was observed at 0.225 g L^−1^ of NaNO_3_ at the 13th day of culture. However, high PE content was detected since the ninth day (10.37 mg L^−1^). Similarly, 10.58 mg L^−1^ PE was determined at 0.075 g L^−1^ of NaNO_3_, but obtained at the 10th day. The PE content was lower (maximum value of 4.29 mg L^−1^) when the concentration of NaNO_3_ was 0.450 g L^−1^. The results of PE concentration under a light intensity of 65 µmol m^−2^ s^−1^ are shown in Figure 2B. Six days of a lag phase were observed when the concentration of NaNO_3_ was set at 0.075 g L^−1^. This experiment showed a long exponential phase of seven days, reaching a maximum PE concentration of 15.18 mg L^−1^ (3.10 mg g^−1^ PE/DW). Lower concentrations of PE (4.74 and 4.71 mg L^−1^) were observed when the concentration of NaNO_3_ was set at 0.225 and 0.450 g L^−1^, respectively, at day 10. The experiments under a light intensity of 100 µmol m^−2^ s^−1^ are shown in Figure 2C. Maximum PE production of 14.65 mg L^−1^ (2.71 mg g^−1^ PE/DW) was obtained at day 14, with NaNO_3_ at 0.075 g L^−1^. When the concentration of NaNO_3_ was 0.450 g L^−1^, it resulted in a maximum PE production of 10.73 mg L^−1^ (4.13 mg g^−1^ PE/DW) after 10 days. A significantly lower value of maximum PE production (4.35 mg L^−1^) was observed with NaNO_3_ concentration of 0.225 g L^−1^. The best value of PE productivity (1.08 mg L^−1^ d^−1^) was observed under a light intensity of 65 µmol m^−2^ s^−1^ and 0.075 g L^−1^ of NaNO_3_. These same conditions were used to obtain the highest PE accumulation.

In addition, the nitrogen and phosphorus ratio was changed between the three experiments. The N:P ratio calculated for each experiment is represented by 11:1, 33:1 and 66:1 respectively with 0.075, 0.225 and 0.450 g L^−1^ concentrations of NaNO_3_. The best PE production is at 11:1 N:P ratio in combination with high light intensity. The higher N:P ratios did not promote PE production, except the 66:1 N:P ratio at the high light intensity with a PE production of 10.73 mg L^−1^ (4.13 mg g^−1^ PE/DW) and the 33:1 ratio at the low light intensity with a PE production of 11.25 mg L^−1^ (5.70 mg g^−1^ PE/DW).

Similar to the biomass behavior, the highest values of PE content were observed under light intensities of 65 and 100 µmol m^−2^ s^−1^ (15.18 and 14.66 mg L^−1^, respectively) that correspond to 3.10 and 2.71 mg g^−1^ PE/DW when the experiments were maintained at 0.075 g L^−1^ of NaNO_3_. PE production under 65 µmol m^−2^ s^−1^ was reduced by 69% and 71% when the concentration of NaNO_3_ was increased to 0.225 and 0.450 mg L^−1^. Under the experiment at 100 µmol m^−2^ s^−1^, a similar effect occurred reducing PE in 70% and 27%, respectively. Experiments under a light intensity of 30 µmol m^−2^ s^−1^ did not affect PE when the nitrate concentration increased from 0.075 to 0.225 g L^−1^.

The best values of PE production were observed in the experiments maintained at 0.075 g L^−1^. An increment of the light intensities of 65 and 100 µmol m^−2^ s^−1^ increased PE production by 43% and 30%, respectively. However, a contrary effect was observed when the concentration of NaNO_3_ was fixed at 0.225 g L^−1^, causing a PE reduction of 57% and 61%, respectively, due to the light intensities of 65 and 100 µmol m^−2^ s^−1^. Experiments with NaNO_3_ at 0.450 g L^−1^ did not show any effect when light intensity increased from 30 to 65 µmol m^−2^ s^−1^. However, when cultures were exposed to 100 µmol m^−2^ s^−1^, PE production increased by 149%.

## 3. Discussion

### 3.1. Mathematical Model Parameter Analysis

The model fitness to the experimental data was high for almost all curves (over 90%). Even the two that were about 80% was good enough considering the biological variability of the system. The Gompertz model helped to accurately estimate the productivity and latency parameters of the *P. purpureum* culture. The mentioned parameters were used to distinguish the kinematics of culture growth and differentiate the actual potential to select the best conditions for biomass production.

Regarding the selection of the best set of culture conditions to maximize biomass, higher productions were found in 65 and 100 µmol m^−2^ s^−1^, compared to 30 µmol m^−2^ s^−1^. For instance, the best NaNO_3_ concentration was 0.075 g L^−1^, with no significant difference (*p* = 0.084) between light intensities of 65 and 100 µmol m^−2^ s^−1^. Although, it is possible that the higher light intensity can be better for larger volume scale with a longer light path.

### 3.2. Effect of Light Intensity on Biomass and PE Production in P. purpureum

The high impact of light intensity on biomass production by *P. purpureum* was evident in this study. Light intensities of 65 and 100 µmol m^−2^ s^−1^ resulted in increases of 253% and 390% of biomass production, respectively, compared to that obtained with 30 µmol m^−2^ s^−1^. However, surprisingly, this increased biomass production was observed when NaNO_3_ was lower than 0.075 g L^−1^. Experiments carried out at 0.225 and 0.450 g L^−1^ of NaNO_3_ also showed lower increments on biomass production (up to 187%) at high light intensities. In the literature, light intensities ranging from 18.75 to 50 µmol m^−2^ s^−1^ resulted in biomass production in *P. purpureum* of up to 0.950 g L^−1^ [36,37,38]. On the other hand, higher levels of light intensity (100 to 140 µmol m^−2^ s^−1^) yielded biomass production above 2 g L^−1^ [26,39]. A study with *Porphyridium marinum* was able to produce 2 g L^−1^ with a light intensity of 150 µmol m^−2^ s^−1^ to later change the condition to 70 µmol m^−2^ s^−1^ [40]. The trends in the present study and in the literature mentioned above indicate that high biomass production occurs at a high light intensity. A similar trend has been observed in *P*. *purpureum* SCS-2 [41] and several other microalgae strains, such as *Chlorella vulgaris* [42], *Parachlorella* sp. [19], and *Spirulina platensis* [43]. It is important to compare these results with other similar biomass yields found in other studies with *P. purpureum* (shown in Table 2). The yield of biomass production by weight is within the range of the presented reports. Our hypothesis for this effect is that low nutrient availability in the culture medium may induce polysaccharide, starch and/or carbohydrate accumulation, [27,44,45,46], and coupled with low light intensity, which promotes the accumulation of lipids 19.4% dry weight [37] and/or dark light cycle [47]. By using the mechanisms of photo-adaption and photo-protection with different light wavelengths sustained 17% dry weight [27]. Guihéneuf and Stengel in 2015 performed a study with several culture conditions, where the nitrogen was maintained replete, limited and depleted. The results of their work show that in nitrogen depleted cultures, *P. purpureum* accumulates carbohydrates (57% dry weight), and interestingly found that the biomass productivity reached 0.15 g L^−1^ d^−1^.

Experiments under low light intensity (30 µmol m^−2^ s^−1^) when the nitrate concentration increased from 0.075 to 0.225 g L^−1^ did not affect PE production. However, 60% of PE decrease was observed as a result of an increment of NaNO_3_ concentration at 0.450 mg L^−1^. A similar effect was observed by Sánchez–Saavedra since they reported a PE production of 44.45 µg L^−1^ under a light intensity of 200 µmol m^−2^ s^−1^ [25]. In contrast, the evaluation at a light intensity of 50 µmol m^−2^ s^−1^ caused a reduction of 60% in the PE concentration. For instance, we can hypothesize that at high light intensity more PE molecules are produced in order to harvest the maximum amount of photons reaching a limit.

It is reported in the literature that an extremely high light intensity causes low biomass yield, due to a phenomenon of photoinhibition in microalgae especially at the beginning of the curve when the culture is non-dense enough [48]. The research done by Guihéneuf and Stengel in 2015, where they explored different conditions to growth *P. purpureum* the best light intensity was at 100 µmol m^−2^ s^−1^ compared to results with 30 and 200 µmol m^−2^ s^−1^, where the biomass and other compounds production were decreased substantially. In accordance with Guihéneuf and Stengel, in the present study, the light intensity of 100 µmol m^−2^ s^−1^ was the highest level evaluated and it resulted in the best biomass yield. Therefore, these results suggest that higher light levels, on the range of evaluation above of 100 µmol m^−2^ s^−1^ could improve biomass yield as a result of the direct relationship between the increase of biomass produced and the light intensity, but at an intensity below 200 µmol m^−2^ s^−1^ similar to the report of Gargouch et al. (2018). This effect may be due to the evaluated strain of *P. purpureum* which showed high photosynthetic efficiency to transform this energy into high biomass production [49]. The photosynthetic efficiency of *P. purpureum* should be related to the accumulation of PE since this molecule is responsible for the light absorption [50]. Furthermore, studies on the macroalgae *Gracilaria domingensis* have shown this trend where high irradiance stimulated the accumulation of proteins [51].

A possibility to select a lower light intensity could arise due to the reduction of cost by using less energy for light in an upscale process. This option could be tested by the cost-benefit analysis of a larger-volume culture set-up against the possible decrease in the penetration of light through it. Moreover, other similar parameters should be tested, such as mixing, aeration, and temperature as is recommended in a previous study [27].

### 3.3. Effect of Nitrogen Concentration on Biomass and Phycoerythrin from P. purpureum

At low light intensities, the biomass production was independent of nitrate concentrations used in this study. At a high light intensity, the systems became sensitive to higher concentrations of nitrate, reducing the biomass yield. In contrast, biomass production was reduced by 50% when the concentration of NaNO_3_ was increased in 0.225 and 0.450 g L^−1^ under a light intensity of 65 and 100 µmol m^−2^ s^−1^. Therefore, the higher biomass production resulted when the lower value of NaNO_3_ was evaluated (0.075 g L^−1^) only when high light intensities were applied. A similar effect is reported in a study done by Kavitha et al. (2016), where they evaluated 1 and 2 g L^−1^ of NaNO_3_ for production of *P. purpureum* biomass. High levels of biomass (3.4–9.4 g L^−1^) from *P. purpureum* were reported by Kathiresan et al. (2007), who suggested that low concentrations of chloride, nitrate, and phosphate did not produce any significant effect on the biomass production. However, an increment of NaNO_3_ above 1 g L^−1^ provokes a negative impact on biomass yield as well as in PE production. It is important to notice, that the main effect caused by a disproportionate increase in the concentration of nitrogen source is the possible limitation of other nutrients, such as phosphorus, trace metals and vitamins [46].

In the present study, increments of the nitrogen concentration were evaluated to obtain higher biomass production and higher PE accumulation. However, under the culture conditions evaluated, the best results were obtained at a low nitrogen concentration (0.075 g L^−1^). Phosphorous is also an important element for microalgae growth, which in conjunction with nitrogen allows biomass production; still, microalgae cannot absorb nitrogen (N) without the presence of phosphorus (P) in the culture, or vice versa, because a proper N:P ratio is essential for their growth [52]. The phosphorous concentration was maintained at 0.005 g L^−1^ (original concentration of the culture medium). The increase of nitrogen concentration changed the N:P ratio. Therefore, results suggest that nitrogen concentration could be increased, but if the N:P ratio of the original culture medium (15:1) is maintained. As studied previously by Razaghi in 2014, the results presented here show similar behavior for N:P ratio change impact the *P. purpureum* growth and PE production. In contrast, the report of Gargouch et al. (2018), where the strain *P. marinum* is used to produce PE (a maximum of 40 mg/g of PE/DW). The culture medium was optimized to produce high biomass and PE and the formulation contains 3.4 g L^−1^ of NaNO_3_ and 0 g L^−1^ K_2_HPO_4_. This reflects a complete different behavior to *P. purpureum* regarding the N:P ratio in terms of this study and others [23,24,37,41,46,52].

## 4. Materials and Methods

### 4.1. Strain, Medium and Culture Conditions

The marine strain *Porphyridium purpureum* UTEX LB 2757 was obtained from the University of Texas, United States of America. The strain was maintained in a mixture (1:1) of Bold 1NV media (UTEX, Weimar, TX, USA) and Erdshreiber media (UTEX, Weimar, TX, USA) in 500 mL flasks containing 300 mL of culture medium. The inoculum (1:10 *v/v*) was cultured at 20 °C in a flask containing 300 mL of culture medium mixed by bubbling sterile air at a flow rate of 25 mL min^−1^. The culture was continuously illuminated with white fluorescent tubes with an average intensity of 65 µmol m^−2^ s^−1^ for 9 days. Then, aliquots of the culture were taken to seed 500 mL Erlenmeyer flasks filled with 270 mL of medium and 30 mL of inoculum coupled with sealed-in tubes to pass a stream of air. Two key factors, the light intensity and nitrogen limitation influencing the production of PE pigment were evaluated. The light intensity was designated as high, medium or low by adjusting different levels of the intensity (100, 65, and 30 µmol m^−2^ s^−1^) with a combined light with wavelengths of green and blue (520–525 and 465–468 nm). The effects of nitrogen concentration were evaluated at three levels of NaNO_3_ concentration (0.450, 0.225 and 0.075 g L^−1^), keeping the F/2 Phosphorous content fixed. Culture nutrient composition was based on the medium F/2 (UTEX, TX, USA), temperature and air flow rate were constant at 20 °C and 25 mL min^−1^. Then, samples were collected each day within 15 days including the days 0 to 3, 6 to 10 and 13 to 14 for the analysis of total biomass and PE produced by microalgae. The microalgae biomass was washed three times with MilliQ water. The samples were centrifuged for 15 min at 4500 rpm. The supernatant was discarded and the pellet was resuspended in MilliQ water. Then, the sample was filtered through a Whatman regenerated cellulose membrane filter, pore size: 0.45 µm, diameter 4.7 cm followed by drying in an oven at 105 °C for 24 h.

### 4.2. Mathematical Model and Kinetic Parameters Calculation to Determine Growth Parameters

In this study, the Gompertz model (Equation (1)) was used to determine maximum specific growth rate (μ_max_, Equation (2)), duration of the latency phase (Equation (3)), duplication time of microalgae cells (Equation (4)) and biomass productivity (Equation (5)) in experiments at different conditions of light intensity and NaNO_3_ concentration.

(1) $$Y=ae^{\left(-e^{\left(b-ct\right)}\right)}$$

where:

$Y=\log\left(N/N_{0}\right)$, $N_{0}$. Initial biomass (t=0) in the sample (mg L^−1^), biomass counts for each time.
t: Time (h).
a: Constant determined from the experimental data. Represents the maximum population of biomass counts when time increases indefinitely.
b: Constant determined from the experimental data.
c: Constant determined from the experimental data.

Maximum specific growth rate:

*μ_max_* = *a* × *c* × (*h* − 1) (2)

Duration of the latency phase:

*λ* = (*b* − 1)/*c* × (*h*) (3)

Generation or duplication time:

*G* = *ln* (*2*)/*μ_max_* (*h*) (4)

The biological parameters determined by the Gompertz model were estimated using the catalog of non-linear regression functions offered by Minitab Inc. 17.1.0 software (LEAD Technologies, Inc., Charlotte, North Carolina, USA) with the Levenberg—Marquardt algorithm.

The determination coefficient (*R*^2^) of linear regression model obtained between the experimental and simulated results allow us to estimate the precision of the experimental data with the model obtained, as well as the quality of the model to replicate the results. Values above 95% conclude that the model manages to replicate 95% of the experiments were carried out with very good quality.

Biomass productivity ($P_{x}$) was calculated (Equation (5)) as follows:

(5) $$P_{x}=\frac{X_{max}-X_{i}}{t_{max}}$$

where $X_{max}$ and $X_{i}$ are maximum and initial biomass concentrations, respectively, and $t_{max}$ is the time corresponding to $X_{max}$.

### 4.3. PE Extraction Methodology

A sample of 2 mL from each *P. purpureum* culture was centrifuged at 4500 rpm for 15 min and the supernatant was discarded. The sediment was mixed with 2 mL of distilled water and was vortexed until homogenization. The suspension was frozen at −20 °C for one hour, and then it was thawed and sonicated in an ice bath for 10 min. The sample was vortexed until homogenization and repeat steps until completing five cycles. After that, the samples were centrifuged at 4500 rpm for 15 min. The pink supernatant was recovered and measured in a spectrophotometer at 564, 592 and 455 nm. The obtained data were substituted in the Beer and Eshel (1985) equation (Equation (6)) to calculate the concentration of PE:

(6) $$PE\;(mg\;mL^{-1})=[(OD_{564nm}-OD_{592nm})-(OD_{455nm}-OD_{592nm})\;0.2]\times 0.12$$

## 5. Conclusions

The comparison between experimental and modeled results shows that the Gompertz model can be used correctly for modeling the *Phorphyridium purpureum* culture within the presented ranges of light intensities and NaNO_3_. This model has the potential to be a simple and powerful tool for the prediction of biomass and PE production. Although, corroboration is needed to include wider ranges of culture conditions.

Light intensity and nitrogen content resulted in strong impact factors to the biomass production of *P. purpureum* and its PE accumulation. The values obtained in the present study are promising since the production is within those reported in the literature, despite a low nitrogen concentration. In addition, this study is the basis for forthcoming optimization and scaling-up studies. The strategy to follow in future experiments to obtain high PE production by *P. purpureum* is to first promote biomass growth by using low nitrogen and high light intensity. After this nitrogen can be increased and a high light intensity can be sustained.

## Figures and Tables

**Table 1:** Kinetic parameters of *P. purpureum* cultivation under different light intensities and NaNO_3_ concentrations (maximum specific growth rate (*µ_max_*), latency phase (*λ*), duplication time of microalgae cells (*G*), determination coefficient (*R*^2^) and productivity).

		Light Intensity (µmol m^−2^ s^−1^)								
		30			65			100		
		NaNO_3_ (g L^−1^)			NaNO_3_ (g L^−1^)			NaNO_3_ (g L^−1^)		
		0.075	0.225	0.45	0.075	0.225	0.45	0.075	0.225	0.45
Gompertz model constants	a	0.74	0.88	2.05	1.33	1.74	1.04	1.59	1.03	2.27
	b	9.3	14.26	1.51	1.76	1.16	3.11	1.42	1.28	1.27
	c	0.07	0.09	0.007	0.013	0.006	0.017	0.009	0.005	0.008
*µ_max_* (d^−1^)		0.05	0.08	0.015	0.018	0.011	0.018	0.015	0.005	0.018
*λ* (h)		122.3	139.1	70.64	56.1	25.72	124.7	45.13	56	34.31
*G* (h)		13.74	8.22	46.96	38.23	62.27	39.37	46.64	45.78	39.2
*R*^2^ (%)		93.83	94.71	95.42	88.6	91.49	86.2	94.82	91.47	95.59
Productivity (mg L^−1^ d^−1^)		173.2	151.8	138.6	349.8	208.2	160.6	386	258	259.9

**Table 2:** Comparison with recent reports of *P. purpureum* biomass growth and yields.

DW (g L^−1^)	N (g L^−1^)	Nitrogen Source	Y (N/DW)	Time (d)	Light Intensity (μmol m^−2^ s^−1^)	Reference
5.54	1.78	KNO_3_	0.045	16	350	[41]
3.4	1	NaNO_3_	0.049	10	100	[23]
9.12	0.97	NaNO_3_	0.018	14	18.85	[24]
3.0–5.403	0.075–0.45	NaNO_3_	0.0023–0.025	14	100	This report
